# Knowledge, use, and disuse of unconventional food plants

**DOI:** 10.1186/s13002-018-0209-8

**Published:** 2018-01-17

**Authors:** Mayana Lacerda Leal, Rubana Palhares Alves, Natalia Hanazaki

**Affiliations:** 1 Universidade Federal de Santa Catarina, Florianópolis, Santa Catarina Brazil; 2 Programa de Pós-graduação em Ecologia, Instituto Nacional de Pesquisas da Amazônia, Manaus, Amazonas Brazil

**Keywords:** ethnobotany, local knowledge, edible plants, PANC, urban foraging

## Abstract

**Background:**

People’s diets are usually restricted to a small number of plant species, even in regions with great diversity. We investigated the knowledge of residents in Ribeirão da Ilha, a district of Florianópolis (Santa Catarina, Brazil), about unconventional food plants (UFP). We report the UFP of the region, the parts used, the methods of processing, and the reasons for reduced use or even lack of use.

**Methods:**

From June 2014 to January 2015, we interviewed 26 long-established residents and made free listings of plant resources in the region. We also did three guided tours, and 24 residents (among the 26) checked pictures of the mentioned plants in order to identify them.

**Results:**

We identified 63 species distributed in 25 botanical families. Half of the species were mentioned only by one informant. The fruit was the most frequently used part (80% of citations), consumed mainly without processing. Among those species, 27% were used exclusively in the past. The residents attributed non-use to the difficulty in locating the plants and loss of interest in the resource.

**Conclusion:**

Urbanization and environmental restrictions contribute to the difficulty of access to UFP. Encouraging residents to continue using UFP is necessary to perpetuate this threatened knowledge, promote a more diversified and healthier diet, stimulate a greater interaction among people and nature, and promote on farm conservation of edible plants.

## Background

Plants have always been part of human life. Historically, human knowledge and use of plants has been guided by practical needs and cultural predilections [1]. Some plant species are widely distributed and their uses are standard, especially food plants and their uses. Food plants are those that have one or more parts or products that can be used as human food [2]. This definition includes plants that are directly consumed and those that furnish oils, spices and condiments used for cooking. It is estimated that there are around 27 thousand plant species with food potential in the world [3]. How many of these plants are used for this purpose is a very complex question. Prescott-Allen and Prescott-Allen [4] estimated that 103 plant species are responsible for 90% of the world food supply. Although this number is underestimated and does not reflect the number of species that are actually used [4], it challenges us to understand the role of factors not covered by these authors’ analysis (e.g., homegardens as food sources), since it is very likely that there is a large number of species with restricted distributions, whose uses are localized or have become neglected.

This group of underutilized plants has received increasing attention, especially as a reaction to the expansion of monocultures, and has received different denominations. Some terms used to refer to them are: “famine foods” [5]; “alternative food plants” [6]; “wild edible plants” [7, 8]; “unconventional vegetables” or “traditional vegetables” [9]; “plants for the future” [10]. Kinupp and Lorenzi [11] consider that the terms used to refer to these food plants have restrictions because they usually contemplate only one plant category (e.g., vegetables, wild, native), which may generate ambiguities and also requires complements. These authors propose the use of another expression that refers to the food species that have one or more parts with food potential and no common use: “unconventional food plants” – UFP (*Plantas Alimentícias Não-Convencionais - PANC*, in Portuguese) [11]. This term also refers to plants that have uncommon processing methods and usually do not have market value or are only commercialized on small scales [11]. Using this broad definition, UFP can include native and exotic plants, and cultivated and spontaneous ones.

Brazil is one of the most biodiverse countries in the world, with a great number of plants with domesticated populations [12, 13]. However, this diversity is rarely exploited and thus fails to contribute to the agricultural development of the country [13]. In addition, many of these species are undergoing genetic erosion, with consequent loss of genetic diversity [12, 13] and loss of associated knowledge about them. A reduction in cultivation and use of other UFP has also been recognized [9]. In the last decades, urbanization has progressively weakened the relationship among humans, land and food cultivation. In this process, traditional agricultural systems lose space to agribusiness [14] and, as a consequence, the dependency on products supplied by the food industry increases, resulting in the decrease of local food consumption, diet changes, and even peoples’ loss of cultural identity [9].

Examining this process under a dynamic perspective, such changes can be associated to the loss of knowledge related to modernization, which was observed elsewhere [see, for example, 15]. Different authors observed the loss of knowledge about plants among communities close to urban centers when compared to isolated ones [15, 16], and related this process to delocalization. Pelto and Pelto [17] used the concept of delocalization to refer to the ever-increasing network of socioeconomic and political interdependency, which affects people’s diets making them more dependent of foods and products coming from distant places through commercial channels. In contrast, another process is recently reported in the literature, showing how urban forests and urban areas with wild edibles are becoming increasing important in people’s diets [18, 19].

The degree of urbanization or the distance to the urban centers can influence the access to food plant resources, and the knowledge and use of these plants [15, 16]. In the Bolivian Amazon, a study about knowledge and use of wild and semi-domesticated plants in two villages with different distances to the market town showed that people from the remote village knew and used more of these plants than those from the more accessible village [20]. In the Brazilian Pantanal, a study of food plants in four communities showed that the residents of the communities further from the city always mentioned more wild edibles, suggesting that the distance to the urban center favored the relations with the environment, increasing the use of wild products for food [21], and we noticed that some of these wild edibles can be considered UFP. Local studies about UFP are important for documenting species with traditional use value, and for stimulating cultural use and conservation, especially in communities facing socioeconomic transformations due to close proximity to urban centers. In this study, our aim was to investigate the knowledge about UFP of residents in Ribeirão da Ilha, a periurban district of Florianópolis, Brazil. We report the UFP species, parts used, processing methods, and the reasons for the reduction of use, and even the abandonment of the use of UFP, and discuss the implications of these observations.

## Methods

### Study area

**Fig. 1:**
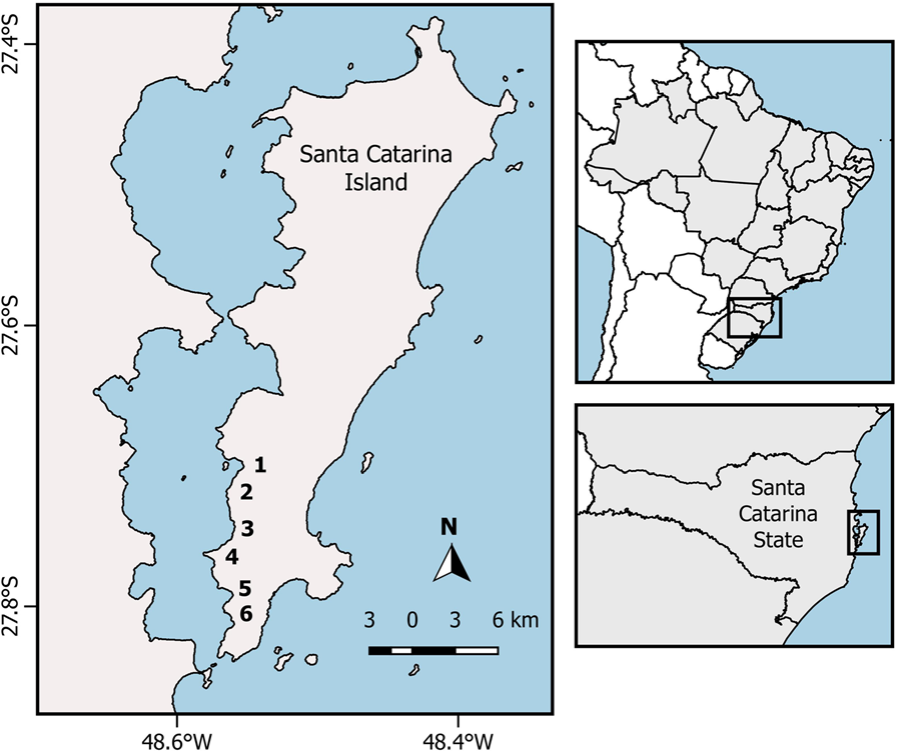
Ribeirão da Ilha district, Santa Catarina Island and communities included in the study. 1 = Alto do Ribeirão, 2 = Ribeirão da Ilha, 3 = Costeira do Ribeirão, 4 = Caiacanga, 5 = Tapera do Ribeirão, 6 = Caieira da Barra do Sul

This study was carried out in the district of Ribeirão da Ilha, located in the southwestern part of Santa Catarina Island, Florianópolis municipality, state of Santa Catarina, Brazil (Fig. 1). The region is located along the coast of the Atlantic Forest, whose original natural vegetation was composed by Dense Ombrophilous Forest [22] and its transition to coastal ecosystems. The study area includes forests, mangroves, beaches, hills and dunes [23]. According to the Köppen classification, Florianópolis’ climate is mesothermal humid, with a hot summer [24]. The relative humidity is 85% and the average rainfall in the south of the city is 1400 mm per year. The average annual temperature is 20 °C along the seafront, where the monthly average temperature is 24 °C in January and 16 °C in July [25].

Ribeirão da Ilha includes eight communities: Carianos, Tapera da Base, Alto Ribeirão, Ribeirão da Ilha, Costa do Ribeirão, Caiacanga, Tapera do Ribeirão and Caieira da Barra do Sul [26]. In this study, we excluded Carianos and Tapera da Base communities, because they are not considered by residents to belong to the district [27]. Until the early twentieth century, agricultural production was intense in this district. Over the following decades, residents left local production to work in the tertiary sector in the growing urban area of the city. In the 1990s, despite having rural features, with small cultivation areas and animal husbandry, the district was considered predominantly residential and focused on domestic tourism [23]. The gradual urbanization of the communities started in the 1990s, and resulted in characteristics combining the maintenance of some rural features (with the presence of small farms and animal husbandry), side by side with an urban arrangement of paved streets, access to public transportation and presence of social services (such as markets, schools, and health centers).

### Data collection

This research was authorized by the Ethics Committee for Research with Human Beings of the Universidade Federal de Santa Catarina – UFSC (32,932,414.8.0000.0121) and interviews were done after the residents signed free informed consent forms. From June 2014 to January 2015, we interviewed residents, who were asked to free list the UFP [28]. These interviews were done with residents selected by the snowball technique [29]. The criteria for inclusion were: (1) to be a long-established resident of the district; (2) to have current or past contact with the local plant resources. No minimum residence time was established to define and select the long-established residents.

First, we conducted five interviews and prepared a species list, covering distinct parts used as food, in order to help in the explanations and to standardize the induction of the free listings of UFP. Based on the results of these preliminary interviews we selected the following species in order to explain what we meant with UFP: root of sorrel (“azedinha”, *Oxalis debilis* Kunth); fruit of Surinam cherry (“pitanga”, *Eugenia uniflora* L.); arrow leaf (“taiá”, *Xanthosoma sagittifolium* (L.) Schott); and nut of queen-palm (“coco-de-cachorro”, *Syagrus romanzoffiana* (Cham.) Glassman). The first part of the interviews addressed the socioeconomic characterization of the family unit: age of respondent, household composition, current occupations, and family income. Then the interviewees were asked to free list the UFP they knew. For each listed plant, we asked about cultivation and plant collection practices; we also asked about ways of obtaining them, parts used, methods of processing, if the use is current or past, and the reasons for the end of the UFP use.

We interviewed 26 residents, among whom 24 participated in the recognition of the species and three in guided tours. The guided tours had an average of 90 min of duration. The interviewees were on average 75 years old, the youngest 56 and the oldest 89 years old. Nine interviewees are male and 17 females. All were born in Santa Catarina and only three of them were not born in Ribeirão da Ilha, but they had lived in the district for at least 40 years. Their main occupations were: retirees or pensioners (*N* = 15 interviewees), housemaids (*N* = 3), work in their own business in the district (*N* = 3), aquaculture (*N* = 2), agriculture (*N* = 2) and general services (*N* = 1).

After all interviews, we identified the species mentioned in the free listings. We showed pictures of the possible species for stimulate recognition by residents [30], and made guided tours and botanical collections [28]. The pictures used were photos taken in situ during the tours, and from [31–34], selected according to their popular names. For very similar species in which the pictures did not allow their recognition (e.g., “tucum”, *Bactris* spp.), the identification was done to the genus. When the popular name covered many species (e.g., several Myrtaceae species), we did a preliminary survey with the interviewees about the plant characteristics to select a smaller number of pictures to be shown. The collected material was herborized and identified using dichotomous keys and with the help of experts. The vouchers were deposited in the FLOR Herbarium (UFSC) and the EAFM Herbarium (Instituto Federal do Amazonas – IFAM), that receive no-fertile material, under the collection numbers from 1 to 18 (M. L. Leal).

### Data analysis

We used descriptive statistics to analyze socioeconomic and UFP data. All species cited in the interviews were considered to characterize the knowledge and the uses of UFP by the residents, except for the species used in the induction of the free listings. We classified the plants as native or exotic to Brazil according to the “Lista de Espécies da Flora do Brasil” [33, 35]. In cases of conflict between the sources, the species were considered exotic because some domesticated exotic species are considered native by “Flora do Brasil” [35].

## Results

Currently 14 interviewees cultivate plants and 23 used to do so in the past, representing a 35% reduction of this practice among them. The reasons that led to the end of cultivation were: environmental ban (*N* = 5 citations), decreased land available (*N* = 4), employment change (*N* = 2), and health problems (*N* = 2). Currently 13 interviewees collect plants and 24 used to do so in the past, representing a 42% reduction of this practice among them. The reasons that led to this change were: resource disappearance (*N* = 5), and loss of interest in the resources (*N* = 4). Other causes mentioned by only one interviewee were: availability of food to purchase (thus there is no need to plant crops), environmental ban, and health problems (forbidding agricultural labor).

Among the 21 interviewees with whom we used the standardized induction, we registered 427 popular names of UFP, with an average of 11 citations of plants per interview (varying from 24 to two). In total, 63 species and six species identified only to genera belonging to 25 botanical families were identified. Half of the species were mentioned only by one informant. The most representative botanical families were: Myrtaceae (*N* = 14 species), Arecaceae (*N* = 8) and Fabaceae (*N* = 7). The most representative species were: *Syagrus romanzoffiana* (*N* = 12 citations), *Eriobotrya japonica* (*N* = 11), *Eugenia uniflora* (*N* = 10) and *Garcinia gardneriana* (*N* = 10). Among the species, 70% are obtained through collection from the forest. Most of them (70%) are native to Brazil and 27% are not currently consumed (Table 1). The reasons for the end of use were: difficulty to find the resource (*N* = 14 citations), loss of interest (*N* = 7), lack of plant attractiveness for consumption (*N* = 7), the absence of the plant in the area or loss of contact with the plant (*N* = 3), plant consumption causes discomfort (*N* = 1), physical limitation for the collection, cultivation and preparation for consumption (*N* = 1).

**Table 1:** Unconventional food plants according residents of Ribeirão da Ilha district, Florianópolis, Brazil (*N* = 21 interviews)

Botanical Family Species	Portuguese name	Part used	Origin	Number of citations
Amaranthaceae				
*Plantago* spp. L.	mastruz	B	N	1
Anacardiaceae				
*Anacardium occidentale* L.	caju	F	N^2^	1
*Spondias dulcis* G. Forst.	cajá-manga	F	E^2^	1
*Spondias mombin* L.*	cajaca, cajá	F	N^2^	1
*Spondias purpurea* L.*	seriguela	F	E	2
Annonaceae				
*Annona glabra* L.*	fruta-conde	F	N	1
*Annona reticulata* L.*	fruto-conde	F	E^2^	2
*Annona neosalicifolia* H. Rainer*	fruta-do-conde-do-mato, curtiça	F	N	1
Arecaceae				
*Bactris setosa* Mart.*	ticum	C	N	1
*Bactris* sp. Jacq. ex Scop.*	tucum, ticum	F, C	E	5
*Butia eriospatha* (Mart. ex Drude) Becc.*	butiá	F, C	N	2
*Butia* sp. (Becc.) Becc.*	butiá	F, C	N	3
*Butia catarinensis* Noblick & Lorenzi	butiá	F, C	N	1
*Euterpe oleracea* Mart.	açaí	F	N^2^	1
*Euterpe edulis* Mart.	coco-de-ripa, açaí	F, C	N	2
Asteraceae				
*Sonchus oleraceus* L.	dente-de-leão	C	E	1
Bixaceae				
*Bixa orellana* L.	urucum	C	N	1
Boraginaceae				
*Varronia polycephala* Lam.*	caramona, mijigrilo	F	N	8
Bromeliaceae				
*Aechmea comata* Baker	bromélia (“chup-chup”), gravatá	L, F, B	N	2
*Ananas bracteatus* Schult.f.	ananá	F	N	1
Cactaceae				
*Cereus jamacaru* DC.*	cacto-cardero, cacto	F	N^2^	1
*Rhipsalis teres* Steud.	olho-de-pinto	F	N	1
Clusiaceae				
*Garcinia brasiliensis* Mart.	bacupari	NA	N^2^	1
*Garcinia gardneriana* (Planch. & Triana) Zappi*	bacopari, bacupari, abricó, damasco	F	N	10
*Mammea americana* L.	biricó, abiricó	F	E	2
Cucurbitaceae				
*Cucumis melo* var. *dudaim* (L.) Naudin	melãozinho	F	E	1
*Momordica charantia* L.	melãozinho	F	E	1
Ebenaceae				
*Diospyros digyna* Jacq.	sapota	F	E	3
*Diospyros inconstans* Jacq.	marmelo	F	N	1
Ericaceae				
*Gaylussacia brasiliensis* Meisn.	camarinha	F	N	1
Fabaceae				
*Inga cinnamomea* Spruce ex Benth.*	angá-banana, angá, ingá	F, C	N^2^	4
*Inga edulis* Mart.*	angá	F	N^2^	2
*Inga marginata* Willd.*	angá-feijão, ingá-feijão, angá	F, C	N	6
*Inga sessilis* Mart.*	angá, angá-macaco, ingá, ingá-macaco	F	N	6
*Inga* sp. Mill.*	angá, angá-banana	F	N	2
*Inga vera* subsp. *affinis* (DC.) T.D. Penn.*	angá, ingá	F	N	2
*Inga vulpina* Mart. ex Benth.*	ingá, angá, ingá-banana	F	N	3
Malpighiaceae				
*Malpighia emarginata* ex DC.*	acerola	F	E	3
Melastomataceae				
*Leandra australis* Cogn.*	mixirica, mixirico	F	N	1
Moraceae				
*Artocarpus heterophyllus* Lam.*	jaca	F	E	5
*Artocarpus integer* (Thunb.) Merr.*	jaca	F	E	1
*Ficus carica* L.*	figo-roxo, figo	F	E	1
*Morus nigra* L.*	amora-do-mato, amorinha, amora, amorinha-do-mato	F	E	9
Myrtaceae				
*Campomanesia adamantium* (Cambess.) O. Berg*	gabiroba	F	N	1
*Campomanesia guaviroba* (DC.) Kiaersk.*	gabiroba	F	N	2
*Eugenia brasiliensis* Lam.*	grumixama	F	N	5
*Eugenia itaguahiensis* Nied.*	grumixama	F	N^2^	1
*Plinia cauliflora* (Mart.) Kausel*	jabuticaba	F	N	5
*Myrciaria glazioviana* (Kiaersk.) G. M. Barroso ex Sobral*	cabeluda	F	N^2^	7
*Myrciaria* sp. O.Berg*	jabuticaba	F	N	7
*Plinia coronata* (Mattos) Mattos*	jabuticaba	F	N^2^	5
*Plinia edulis* (Vell.) Sobral*	cambucá	F	N	8
*Psidium cattleianum* Sabine*^1^	araçá, araçá-amarelo, araçá-roxo, araçá-vermelho, araçá-manteiga	F	N	7
*Psidium grandifolium* Mart. ex DC.*	araçá-cavalo	F	N	1
*Syzygium cumini* (L.) Skeels*	jambolão, jambo	F	E	1
*Syzygium jambos* (L.) Alston*	jambo	F	E	6
Passifloraceae				
*Passiflora edulis* Sims*	maracujá-roxo	F	N	1
Poaceae				
*Cymbopogon citratus* (DC.) Stapf*	capim-limão	L	E	1
Rosaceae				
*Eriobotrya japonica* (Thunb.) Lindl.*	ameixa-amarela	F	E	11
*Rubus idaeus* L.*	framboesa	F	E	1
*Rubus rosifolius* Sm.*	maranguinho-do-mato	F	N	3
*Rubus sellowii* Cham. & Schldtl.*	amora-do-mato, amorinha, amorinha-do-mato	F	N	5
Sapotaceae				
*Mimusops commersonii* Engl.	biricó	F	E^2^	1
*Pouteria caimito* Radlk.	abiu	F	N^2^	1
Solanaceae				
*Solanum aculeatissimum* Jacq.*	rebenta-cavalo	F	N	1
Tropaeolaceae				
*Tropaeolum majus* L.	capuchinha	B	E	1

Part used: stem (S), flower (B), leaf (L), fruit (F), root (R), seed (C). Plant origin: native (N) or exotic (E) to Brazil. No data available (NA). *The currently used species were marked with an asterisk

^1^The interviewees considered these as different plants, but they belong to the same botanical species

^2^These species do not occur naturally in the region, and were probably cultivated by the residents

The fruit was the most commonly used part (*N* = 313 citations), followed by the seed (*N* = 36), root (*N* = 17), leaf (*N* = 11), flower (*N* = 2) and stem (*N* = 1) (Table 1). We did not include in Table 1 the species used during induction of the interviews (*Oxalis debilis, Eugenia uniflora*, *Xanthosoma sagittifolium*, and *Syagrus romanzoffiana*), but they were also mentioned in the interviews for other uses not exemplified in the induction (e.g., consumption of leaves of *O. debilis* and *E. uniflora* used to make sauces). Consumption without processing was the most cited use (*N* = 57). The other ways of consumption were: as juices, *Spondias purpurea*, *Cymbopogon citratus, Plantago* spp., *Butia eriospatha*, *Butia* sp., *Euterpe edulis*, *Malpighia emarginata*, *Morus nigra*, *Eugenia uniflora*, *Passiflora edulis*, *Rubus idaeus*, *R. rosifolius*, *R. sellowii*; in alcoholic beverages, *Butia catarinensis*, *Butia* sp., *E. brasiliensis, Rubus sellowi, Myrciaria* sp., *Morus nigra*; to make sweet sauces, *Eugenia uniflora*, *Rubus sellowii, Malpighia emarginata, Morus nigra*; to make jams, *Plinia cauliflora*, *P. coronata*, *Malpighia emarginata*; in salads, *Sonchus oleraceus* and *Tropaeolum majus*; to make crumbs (“farofa”); preparation involves the maceration of a seed (*Bactris setosa*) and a fruit (*Varronia polycephala*) with cassava (*Manihot esculenta*) flour; and food coloring (“colorau”) made with the aril of the seed of *Bixa orellana.*

## Discussion

The definition of UFP, as well as the other expressions that can replace it, such as wild edible, or alternative food plant, requires elaboration for better understanding. By classifying a species as UFP, it is necessary to consider the scale of analysis and the group of people who use it. In Ribeirão da Ilha we found species with very restricted uses, such as “olho-de-pinto” (*Rhipsalis teres*), whose use as food was not found in other ethnobotanical studies. On the other hand, we also classified as UFP species some widely known foods, such as Surinam cherry (*Eugenia uniflora*) [36], but that have limited commercial use in the region. Other plants considered here as UFP would not compose the UFP list in other geographical and sociocultural contexts, such as “butiá” (*Butia catarinensis*), cashew (*Anacardium occidentale*) and assai (*Euterpe oleracea*). “Butiá” fruits are used in the production of popsicles and ice creams, and are sold in some localities of the state of Santa Catarina [37]; cashew nuts, fruits and juices are found in different markets of Brazil [38], and assai pulp can even be found in markets outside of the country (e.g., Los Angeles or New York, USA). However, the residents of Ribeirão da Ilha do not make frequent use of these plants, nor do they have easy access to the products mentioned. The knowledge about UFP is difficult to access and is little shared. The difficulty in identifying the species, which required detailed explanations and descriptions of them, shows that they are not really plants of general and recurrent use [11]. In addition, half of the species of this study was cited by only one interviewee. This idiosyncratic knowledge about plants in general, regardless of it being an UFP, is frequent in ethnobotanical studies e.g. [39, 40]. In studies about UFP this knowledge is critical to their identification and the frequency of citation does not necessary reflect the species’ importance.

The most representative families in this study, with a greater number of species cited, were Myrtaceae, Arecaceae and Fabaceae. These families are also highlighted in other studies about general food plants of the Atlantic Forest [36]. The Myrtaceae and Arecaceae families also stand out among the most cited in a study about UFP used by farmers in Rio Grande do Sul, Brazil [41]. Myrtaceae is one of the largest families of Brazil’s flora, with 23 genera and about 1000 species, with the great nutritional value of its fruits a special feature [42]. In Brazil, Arecaceae includes about 40 genera and 200 species, some with major food and ornamental uses [42]. Fabaceae in Brazil includes about 200 genera and 1500 species, covering different categories of use in addition to food, such as timber, firewood, green manure and urban forestry [42].

Most of the identified species are native to Brazil. Some have already been recognized and listed as species of the southern Brazilian region with current or potential economic value to be better exploited, or “plants for the future” [10]. The Surinam cherry (*Eugenia uniflora*), “butiá” palms (*Butia eriospatha* and *B. catarinensis*), “camarinha” (*Gaylussacia brasiliensis*), “grumixama” (*Eugenia brasiliensis*), strawberry guava (*Psidium cattleianum*) and roseleaf bramble (*Rubus rosifolius*) have their food use potential highlighted; the queen-palm (*Syagrus romanzoffiana*) was mentioned because of food and ornamental uses; and “ingás” (*Inga edulis*, *I. marginata*, *I. sessilis*) were considered as bee forage for honey production [10].

Although we identified numerous UFP species, the parts consumed and processing methods were not very innovative. As in other studies, the fruit was the most commonly used part [36, 43, 44], generally consumed without processing. Fruits and vegetables are rich in water, which makes them quite perishable [38]. In a study of 14 native edible plants of the Caatinga, in the Brazilian northeast, nine of them were consumed exclusively *in natura* [45]. In addition, the authors reported that the loss of traditional plant uses also resulted in lost of associated knowledge about their preparation [45]. Ribeirão da Ilha is probably facing a similar situation, as we reported reduction in use of UFP and disinterest by the interviewees in consuming them. In addition, we should consider the age of the interviewees as a factor that contributes to them not remembering unusual practices and other methods of preparation of such plants.

Beyond the low variation in preparation practices, we also reported a reduction in the number of species used, mainly of the idiosyncratic ones. Among the reasons mentioned about the end of use is the difficulty of finding the plants and the loss of interest in them. The process of urbanization of the Ribeirão da Ilha occurred in the last decades [23] and the application of restrictive environmental laws, especially those related to the prohibition of plantations on hilltops [46], probably contributed to the disinterest and the abandonment of agricultural and extractive activities in the district. The absence of these activities, along with the increase of the number of houses and fences, contributes to the real or apparent reduction in the use of UFP. For other localities on Santa Catarina Island with a gradient of urbanization, a difference in the diversity of homegarden plants has been reported for the most urbanized neighborhood when compared to rural and periurban areas [47]. Rural homegardens presented greater floristic diversity in comparison to periurban ones; and there was no difference among periurban and urban homegardens [47]. The richness found in the urban and periurban homegardens was higher than we found in this study (71 and 72, respectively, compared to 63), but the urban homegardens presented less species (54), even our study being restricted to UFP. A few of these species were the same, showing different sets of plants: those kept in homegardens [47] and those that can still be accessed from both homegardens, old crop fields, and other urban areas and are currently considered as UFP. In Ribeirão da Ilha, an area that has the same historical and cultural contexts of the areas studied by Peroni et al. [47], the urbanization process probably influenced the diversity and abundance of edible plants cultivated, especially UFP. The reduction in the areas of cultivation can result in a greater rigor in the selection of the crops, with elimination of little used ones and less space for spontaneous plants. In addition, a more urbanized livelihood and increased access to industrialized products can also contribute to the reduction or lack of interest in autochthonous resources. These changes agree with the process of food delocalization [17], which occurs as the proximity to urban areas and markets increases [16], and that the old ways of life are replaced by modern livelihoods.

## Conclusions

Although government agencies suggest that some UFP are plants for the future [10, 11], in our study we found that in Ribeirão da Ilha some of them are in disuse and knowledge about them is threatened. The barriers and challenges for the integration of UFP in the market involves questions about production, storage and processing, organization of their supply chains, and negative images of their consumption, sometimes correlated with poverty and low social status [38]. Among other things, the popularization of UFP use requires investments in local economies and the creation of consumer markets. Movements such as “Slow Food”, that seek to counteract the current lifestyle with its fast foods, valuing local cultures and food traditions, are obtaining followers worldwide. Such a move is consistent with the pursuit of food security, which, according to the Brazilian food and nutritional security law, says that every human being "[...] has the right to a healthy, affordable diet, with quality and in sufficient quantities, on a permanent basis" [our translation, Federal Law n° 11.346, [48]], based on food practices that promote health without compromising other essential needs. This law emphasizes that food security also requires respect for the sovereignty of the people, a concept that involves respect for the uniqueness and cultural characteristics of each region. This discussion converges to Poe and colleagues’ [18] arguments about urban foraging, which is a legitimate social benefit related to plant collection in urban areas, including a vast array of non-timber forest products. Urban spaces providing plants for food and for other uses comprise ecologically and spatially heterogeneous sites including parks, wooded forests, yards, sidewalks, planting strips on street edges, trails, landscaping areas, empty lots, and abandoned properties [18] and several of them are present at Ribeirão da Ilha. Although widespread in urban areas around the world [19], the information about the use of collected plants for food in urban areas is still scarce. The popularization and valorization of UFP in urban or urbanized areas can contribute to the arguments of urban forest justice, which recognizes the rights of local people to have control over their own culturally appropriate wild food and health systems [18], and also contributing to the valorization of local knowledge and skills.

In Brazil, although still modest, we observe a growing demand for organic and regional foods. The Ministry of Agriculture has a national catalog of organic food producers and provides certification (SisOrg seal), attesting the food’s quality [49]. Related to UFP, we highlight actions to stimulate the consumption and the perpetuation of knowledge, such as offering courses and the recent publication of a book about these plants, which has detailed recipes of a great number of species [11]. Public awareness of the importance of consuming healthier foods is central to creating new market logic, and increasing the investments and consumption of UFP. In areas that have been undergoing increased urbanization, such as Ribeirão da Ilha, the incentive to use UFP is especially important, and is related to the perpetuation of traditional knowledge, which is often threatened. In addition, the increase of use and appreciation of local plant resources can stimulate greater interaction among people and nature and the maintenance of green areas, promoting of on farm conservation of edible plants.

## Abbreviations

IFAM: Instituto Federal do Amazonas
PANC: Plantas Alimentícias Não-Convencionais
UFP: Unconventional Food Plants
UFSC: Universidade Federal de Santa Catarina
